# Discomfort-related changes of call rate and acoustic variables of ultrasonic vocalizations in adult yellow steppe lemmings *Eolagurus luteus*

**DOI:** 10.1038/s41598-021-94489-7

**Published:** 2021-07-22

**Authors:** Anna V. Klenova, Ilya A. Volodin, Olga G. Ilchenko, Elena V. Volodina

**Affiliations:** 1 Department of Vertebrate Zoology, Faculty of Biology, Lomonosov Moscow State University, Vorobievy Gory, 1/12, Moscow, 119234 Russia; 2 Department of Behaviour and Behavioural Ecology of Mammals, A.N. Severtsov Institute of Ecology and Evolution, Russian Academy of Sciences, Leninskii Prospect, 33, Moscow, 119071 Russia; 3 Department of Small Mammals, Moscow Zoo, B. Gruzinskaya, 1, Moscow, 123242 Russia

**Keywords:** Animal behaviour, Animal disease models

## Abstract

Potential of ultrasonic vocalizations (USVs) to reflect a degree of discomfort of a caller is mostly investigated in laboratory rats and mice but poorly known in other rodents. We examined 36 (19 male, 17 female) adult yellow steppe lemmings *Eolagurus luteus* for presence of USVs during 8-min experimental trials including 2-min test stages of increasing discomfort: isolation, touch, handling and body measure. We found that 33 of 36 individuals vocalized at isolation stage, i.e., without any human impact. For 14 (6 male and 8 female) individuals, a repeated measures approach revealed that increasing discomfort from isolation to handling stages resulted in increase of call power quartiles and fundamental frequency, whereas call rate remained unchanged. We discuss that, in adult yellow steppe lemmings, the discomfort-related changes of USV fundamental frequency and power variables follow the same common rule as the audible calls of most mammals, whereas call rate shows a different trend. These data contribute to research focused on searching the universal acoustic cues to discomfort in mammalian USVs.

## Introduction

Human-audible (below 20 kHz) calls of mammalian callers reflect the increase of discomfort universally across species in the higher call rate, the higher fundamental and peak frequencies and the higher power quartiles^1^. This common rule acts across contexts and taxa, from rodents to humans^1–5^.

However, many mammals (primarily rodents and small primates) preferentially use the ultrasonic range of frequencies (over 20 kHz) for social communication^6,7^. As audible calls, the ultrasonic vocalizations (USVs) reflect the degree of negative or positive arousal, thus providing to neuropsychologists the window into processes that determine the course of development, emotional functions and dysfunctions^6^. Example studies are investigations of rat 22-kHz USVs related to the negative contexts/emotions and 50-kHz USVs related to the positive contexts/emotions^8^ and the development of theory of ethotransmitters considering the immediate effects of USVs on the emotional status of recipients^9^.

However, few studies involving rodents aside mice and rats provide direct data of how the acoustic structure and USV variables change with increase of discomfort^10^. Most studies only estimate one single variable (call rate) and only in one age class (in pups)^11,12^. The reason of this is the simplicity of the underlying experimental procedure: pup rodents of all species produce USVs when isolated from warm nest because of their imperfect thermoregulation^13^. Only in some rodent species, short-term isolation of adults at unfamiliar territory is effective for inducing USVs^14^. The yellow steppe lemming *Eolagurus luteus* is one of few species in which USVs can be induced easily in any age and sex in the unified situation of novelty, of the placement of an animal alone in a small new cage^14^.

The yellow steppe lemming is a diurnal Arvicolinae rodent species of about 100 g body weight in either sex. Natural populations of this species inhabit steppes of Mongolia, North-Eastern China, Eastern Kazakhstan and Southern Altai in Russia^14,15^. The yellow steppe lemming potentially represents a very perspective wild-type rodent model for biomedical research including USVs^14^. This species easily breeds and lives in captivity, what enabled recording the audible calls of adults^16^ and tracking the ontogeny of USV acoustic variables^14^. However, the effects of different experimental manipulations and degree of discomfort on the emission probability, call rates and the acoustics of USVs have yet to be studied in yellow steppe lemming.

In contrast to infant pup rodents, for which isolation from the nest induces a stronger discomfort compared to human handling^10,11^, for adult rodents human handling (e.g., hand restraint) might be a more unpleasant stimulus compared to short-term isolation on unfamiliar territory. In this study, we expected that adult yellow steppe lemmings would experience an increasing discomfort during the four successive 2-min stages of experimental trial: isolation, touch, handling and body measure. We expected that in adult yellow steppe lemmings, the elevated discomfort would be associated with a higher call rate and with the energy shift towards higher frequencies of USVs, that is, in the same direction of changes as in the audible calls of mammals^1^. We also expected, based on the findings of a previous study^14^ that subject animals will be highly vocal at all experimental stages, including isolation on an unfamiliar territory.

In this study we compare, by using a repeated measures approach, the call rates and the acoustics of USVs produced by adult individual yellow steppe lemmings during the isolation stage (reflecting basic discomfort) and the handling stage (reflecting elevated discomfort). In addition, we estimate percent of USV callers/non-callers at each of the four stages of the experimental trial.

## Results

### Vocal activity across experimental stages

Inspection of spectrograms showed that 100% individuals vocalized in USV range during the experimental trials (Table 1). Most intense USV calling occurred at isolation stage, at which 33 of the 36 subject individuals produced USVs and 20 of them produced over 50 USVs (two individuals produced as much as 574 and 575 USVs). At touch stage, the USV calling was only slightly less intense: 26 of 36 callers provided USVs and 15 of them produced more than 50 USVs. Further increase of discomfort towards the handling and measure stages resulted in reduced emission of USVs (Table 1). Only 6 of 36 callers provided over 10 USVs at each of the four experimental stages.

**Table 1:** Numbers of USV callers with different vocal activity which provided USVs at different stages of the 36 experimental trials (one trial per animal).

Number of USVs	Trial stage				Number of callers at all trial stages
	Isolation	Touch	Handling	Measure	
0	3	10	12	10	0
1–10	7	6	9	16	6
10–50	6	5	9	6	7
Over 50	20	15	6	4	23
Number of callers at stage	33	26	24	26	36

### Discomfort-related changes of USV acoustic variables

For joint USVs, repeated measures ANOVA showed that the increase of discomfort from isolation to handling stages resulted in the respective increase of all the three power quartiles. The peak frequency of joint USVs and USV call rate did not change between stages (Table 2).

**Table 2:** Values (mean ± *SD*) of USV call rate and of the acoustic variables of joint USVs and repeated measures ANOVA results for comparison between isolation and handling experimental stages.

Acoustic variable	Isolation stage, *N* = 14; *n* = 14	Handling stage, *N* = 14; *n* = 14	ANOVA
USV call rate	2.10 ± 1.57	1.50 ± 1.61	*F*_1,13_ = 1.13; *p* = 0.31
fpeak (kHz)	32.26 ± 3.03	33.16 ± 3.65	*F*_1,13_ = 1.15; *p* = 0.30
q25 (kHz)	29.25 ± 2.06	31.06 ± 2.70	*F*_1,13_ = 11.12; ***p* = 0.005**
q50 (kHz)	32.94 ± 2.74	36.07 ± 1.98	*F*_1,13_ = 28.98; ***p* < 0.001**
q75 (kHz)	37.09 ± 2.33	42.59 ± 2.68	*F*_1,13_ = 46.33; ***p* < 0.001**

Designations: *N*—the number of individuals; *n*—the number of joint USVs; USV call rate—the number of USVs per second; fpeak—the frequency of maximum amplitude; q25, q50, q75—the lower, the medium and the upper quartiles. Significant values are marked in bold.

For single USVs, we estimated the effect of discomfort increase between isolation and handling stages on the acoustics for three different samples: (1) for the total sample of single USVs (with and without frequency jumps), (2) separately for single USVs with frequency jumps and (3) separately for single USVs with continuous contour without frequency jumps. Single USVs with and without frequency jumps were present in all the 14 individuals at both isolation and handling stages.

For the total sample of single USVs (with and without frequency jumps, Table 3), ANOVA showed that the increase of discomfort between isolation and handling stages resulted in respective increase of the maximum, minimum and end fundamental frequencies as well as of the depth of frequency modulation, peak frequency and all the three power quartiles. The beginning fundamental frequency did not change with increase of discomfort. Call duration has shortened (Table 3).

**Table 3:** Values (mean ± *SD*) of the acoustic variables of single USVs and two-way ANOVA results for comparison between isolation and handling experimental stages.

Acoustic variable	Isolation stage, *N* = 14; *n* = 279	Handling stage, *N* = 14; *n* = 280	ANOVA
Duration (ms)	30.00 ± 8.64	26.97 ± 8.93	*F*_1,544_ = 21.62; ***p* < 0.001**
f0max (kHz)	37.55 ± 6.20	43.59 ± 9.17	*F*_1,544_ = 100.27; ***p* < 0.001**
f0min (kHz)	25.17 ± 4.82	26.02 ± 5.90	*F*_1,544_ = 4.52; ***p* = 0.034**
f0beg (kHz)	27.10 ± 6.51	27.29 ± 6.91	*F*_1,544_ = 0.14; *p* = 0.71
f0end (kHz)	34.12 ± 5.57	39.28 ± 8.02	*F*_1,544_ = 101.21; ***p* < 0.001**
df0 (kHz)	12.38 ± 5.50	17.57 ± 7.95	*F*_1,544_ = 86.40; ***p* < 0.001**
fpeak (kHz)	31.36 ± 4.47	34.75 ± 5.23	*F*_1,544_ = 85.53; ***p* < 0.001**
q25 (kHz)	29.92 ± 3.70	32.35 ± 4.03	*F*_1,544_ = 79.14; ***p* < 0.001**
q50 (kHz)	32.60 ± 3.90	35.74 ± 4.50	*F*_1,544_ = 108.19; ***p* < 0.001**
q75 (kHz)	37.28 ± 6.15	41.48 ± 7.26	*F*_1,544_ = 71.90; ***p* < 0.001**

A total sample of USVs (with and without frequency jumps) was used. Animal individual identity was introduced in model as random factor. Designations: *N*—the number of individuals; *n*—the number of single USVs; f0max—the maximum fundamental frequency; f0min—the minimum fundamental frequency; f0beg—the fundamental frequency at the onset of a call; f0end—the fundamental frequency at the end of a call; df0—the depth of frequency modulation; fpeak—the frequency of maximum amplitude; q25, q50, q75—the lower, medium and upper quartiles. Significant values are marked in bold.

Separately for single USVs without frequency jumps (Table 4), ANOVA showed that the increase of discomfort between isolation and handling stages resulted in respective increase of the maximum, minimum, beginning and end fundamental frequencies as well as of the depth of frequency modulation, peak frequency and all the three power quartiles. Call duration has shortened (Table 4).

**Table 4:** Values (mean ± *SD*) of the acoustic variables of single USVs without frequency jump and two-way ANOVA results for comparison between isolation and handling experimental stages.

Acoustic variable	Isolation stage, *N* = 14; *n* = 138	Handling stage, *N* = 14; *n* = 120	ANOVA
Duration (ms)	28.60 ± 9.14	25.72 ± 8.83	*F*_1,243_ = 6.96; ***p* = 0.009**
f0max (kHz)	34.21 ± 5.70	39.01 ± 8.55	*F*_1,243_ = 39.48; ***p* < 0.001**
f0min (kHz)	24.50 ± 5.64	26.12 ± 7.04	*F*_1,243_ = 12.86; ***p* < 0.001**
f0beg (kHz)	24.96 ± 6.00	26.82 ± 7.72	*F*_1,243_ = 11.88; ***p* < 0.001**
f0end (kHz)	33.56 ± 5.93	37.79 ± 8.60	*F*_1,243_ = 29.65; ***p* < 0.001**
df0 (kHz)	9.72 ± 4.80	12.89 ± 7.08	*F*_1,243_ = 14.22; ***p* < 0.001**
fpeak (kHz)	30.82 ± 5.10	34.97 ± 6.41	*F*_1,243_ = 46.10; ***p* < 0.001**
q25 (kHz)	29.33 ± 4.33	31.95 ± 4.63	*F*_1,243_ = 39.17; ***p* < 0.001**
q50 (kHz)	31.80 ± 4.47	35.31 ± 5.66	*F*_1,243_ = 43.18; ***p* < 0.001**
q75 (kHz)	36.80 ± 7.52	41.28 ± 9.12	*F*_1,243_ = 22.29; ***p* < 0.001**

Animal individual identity was introduced in model as random factor. Designations as in Table 3.

Separately for single USVs with frequency jumps (Table 5), ANOVA showed that the increase of discomfort between isolation and handling stages resulted in respective increase of the maximum and end fundamental frequencies as well as of the depth of frequency modulation, peak frequency and all the three power quartiles. Call duration has shortened. Distinctively from the USV sample without frequency jumps, the minimum f0 did not increase and the beginning f0 significantly decreased between stages (Table 5).

**Table 5:** Values (mean ± *SD*) of the acoustic variables of single USVs with frequency jump and two-way ANOVA results for comparison between isolation and handling experimental stages.

Acoustic variable	Isolation stage, *N* = 14; *n* = 141	Handling stage, *N* = 14; *n* = 160	ANOVA
Duration (ms)	31.38 ± 7.91	27.91 ± 8.91	*F*_1,286_ = 22.26; ***p* < 0.001**
f0max (kHz)	40.82 ± 4.77	47.03 ± 8.07	*F*_1,286_ = 57.18; ***p* < 0.001**
f0min (kHz)	25.84 ± 3.75	25.95 ± 4.90	*F*_1,286_ = 0.82; *p* = 0.37
f0beg (kHz)	29.19 ± 6.32	27.65 ± 6.23	*F*_1,286_ = 8.66; ***p* = 0.004**
f0end (kHz)	34.67 ± 5.17	40.39 ± 7.40	*F*_1,286_ = 84.99; ***p* < 0.001**
df0 (kHz)	14.99 ± 4.60	21.08 ± 6.70	*F*_1,286_ = 81.56; ***p* < 0.001**
fpeak (kHz)	31.89 ± 3.69	34.58 ± 4.13	*F*_1,286_ = 39.12; ***p* < 0.001**
q25 (kHz)	30.49 ± 2.85	32.65 ± 3.50	*F*_1,286_ = 40.86; ***p* < 0.001**
q50 (kHz)	33.38 ± 3.07	36.06 ± 3.36	*F*_1,286_ = 65.46; ***p* < 0.001**
q75 (kHz)	37.74 ± 4.38	41.63 ± 5.51	*F*_1,286_ = 58.82; ***p* < 0.001**

Animal individual identity was introduced in model as random factor. Designations as in Table 3.

So, effect of presence/absence of frequency jumps of USVs contour on discomfort-related changes of USVs was weak, as it affected only the minimum and beginning fundamental frequencies. All other measured acoustic variables of USVs were unaffected and changed uniformly in USVs with or without frequency jumps.

## Discussion

This experimental study showed that the values of USV acoustic variables, the fundamental and peak frequencies and all the three power quartiles increased whereas duration shortened from the isolation to handling experimental stages. Probably, these changes were related to increase of negative emotional arousal in subject adult yellow steppe lemmings. Similar trends of changes were found in both nonmanipulated single USVs and in the artificially created joint USVs (prepared by cut of silence spaces between single USVs). Earlier, the isolation context was considered as inducing less discomfort state compared to handling context in the studies of the audible calls of the young of artiodactyls^5^ and felids^17^ and in the study of the ultrasonic calls of laboratory mice pups^11^. In this study of adult yellow steppe lemmings, the applied handling (restraint in human hand with belly up) was more discomfort for animals than isolation. We infer this based on animal attempts to escape from the human hand or to bite. Similar stressing impact of handling on adults was also reported for other species of rodents^18–20^.

Shift of fundamental frequency and power variables toward higher values represents the universal cross-species indicator of increased emotional arousal in the audible mammalian calls^1–3,5^. The same shift of fundamental frequency and power variables toward higher values was found here in USV variables of adult yellow steppe lemmings. This universality of vocal indicators of emotional arousal in audible and ultrasonic calls is surprising because of different mechanisms of sound production for the audible and ultrasonic calls. The audible calls are produced by vibration of the vocal folds^21–23^ whereas the USVs are produced with aerodynamic whistle mechanism^24–26^.

Moreover, these different sound production mechanisms provide similar trajectories of changes with increase of discomfort in spite of the strongly detached frequency ranges. In the audible calls, the fundamental frequency may increase if elevated discomfort triggers some vocal fold changes, as e.g., tension due to increased tissue stiffness or shortening the part of oscillating tissue^27^. The power variables may increase due to increased muscle tension and shrinking of supralaryngeal cavities, shifting the emphasized frequency bands upward^1,28^. Further research is necessary to determine whether the same physiological mechanisms underly the changes of USV parameters at increasing discomfort.

Against expectations, call rate, representing the most commonly used parameter for analysis of discomfort-related changes of USVs in pup rodents (for example^11,12^), did not display the change with increase of discomfort in adult yellow steppe lemmings. However, for rodent pups, the published results regarding the changes of call rate are rather controversial even within species. For example, for pup laboratory mice, the study by^11^ showed that call rates for ultrasonic calls are higher during rotation of handheld pups than during isolation from the 2nd to the 8th day of pup life. At the same time, the study by^29^ showed that call rates are lower during handling than during isolation on the 8th day of life, but indistinguishable on the 4th day of life. Accordingly, in 10–18-day old pup fat-tailed gerbils *Pachyuromys duprasi*, USV call rate was higher at isolation than at handling, whereas in the older 20–40-day old pups, call rates did not differ between isolation and handling stages^10^.

This study investigated also the methodical point of the effects of presence/absence of frequency jumps on measurements of discomfort-related acoustic variables. This brief investigation is important for further research of USV variables in rodents, because frequency jumps are enormously widespread in pup and adult rodent USVs^6,9,14,30–32^. This most frequent nonlinear phenomenon affects very strongly the values of USV fundamental frequency. However, surprisingly, it did not affect the changes of values of the acoustic variables under the increasing discomfort in yellow steppe lemmings. Therefore, we conclude that presence/absence of frequency jumps can be ignored by further studies of discomfort-related changes of USVs in yellow steppe lemmings and probably also in similar studies of USVs in other rodents.

We also estimated the use of joint USVs for comparison of the acoustic variables between the different experimental stages within individual, following^2,3,10^. Joint USVs enable measuring the acoustic variabes of all USVs produced by a focal caller within a certain time period. This allows avoiding a problem of selection of calls for acoustic analysis, which arises with single USVs because single USVs can be strongly different within individual. Furthermore, this way of measuring of acoustic variables is less laborious compared to the analysis of the acoustic variables of single USVs and can be easily automatised.

Vocal correlates of emotional arousal in the audible calls have proved to be useful indicators of discomfort and welfare^33–36^. However, little is known about the vocal correlates of emotional arousal in USVs of mammals^8,10^. The varying contexts and vocalizing thresholds in different rodent species complicate comparison of the degree of emotional arousal and makes difficult the search of the universal cues of increasing discomfort in USVs. At the same time, USVs represent the most frequent type of vocal activity in laboratory rodents, including animal models of human disorders and diseases^6,37,38^. The high vocal activity of all age and sex-class yellow steppe lemmings in isolation makes this species a promising wild-type animal model for biomedical research^14^. In this study, most animals produced USVs at isolation on unfamiliar territory without any additional manipulations. The high emission of USVs by adult rodents isolated in a setup is rare advantage, promoting the use of yellow steppe lemming for modelling diseases and testing the effects of drugs.

## Methods

### Animals and housing

The USVs were collected during experimental trials from 36 adult yellow steppe lemmings (19 males and 17 females with breeding experience) at Moscow Zoo, Moscow, Russia, between February 02–July 30, 2018 and in March 16–19, 2020. All subjects were captive-born descendants of 7 individuals, obtained by Moscow Zoo in autumn 2016–spring 2017 from a natural population in East Kazakhstan (48°10′N, 84°25′E). The animals were kept in pairs with or without pups under a natural light regime at room temperature 22–25 °C, in wire-and-glass cages of 50 × 100 × 35 cm with a bedding of sawdust (for housing details, see^14^). Pregnant females did not participate in the experiments.

### Experimental procedure and USV recording

Experimental trials were made in a room where no other animals were present, at room temperature 22–25 °C during daytime, at the same level of background noise. Experimental procedure followed^39^. The focal animal was taken from its home cage before the test trial with a clean plastic glass and returned to the cage after the test trial. Each animal was tested singly in one 8-min experimental trial, including the four 2-min successive stages: isolation, touch, handling and body measure. The trial started, when the focal animal was placed to the experimental setup (the open from above plastic cylinder without bottom of diameter 193 mm, high 170 mm standing on smooth table surface). During isolation stage, the animal was unrestrained. During touch stage, the animal was gently touched by experimenter (IAV) with a cotton bud, approximately two times per second. During handling stage, the animal was handheld being rotated on its back. During body measure stage, the experimenter thrice measured body length, head length, foot length the tail length with electronic calipers. The end of measurements was the end of the trial. The measuring data were used in^14^ and not used in this study. After each trial, the experimental setup was washed with soapy water and rubbed with napkin wetted with alcohol to avoid effect of smell on USV of the next focal animal in the next experimental trial^40^.

For USV recording (sampling rate 384 kHz, 16 bit resolution) we used a Pettersson D1000X recorder with built-in microphone (Pettersson Electronik AB, Uppsala, Sweden). The microphone was established stationary at distance 35 cm above the cylinder with animal. The obtained recordings had a high signal/noise ratio, the reverberation practically lacked. Each trial was recorded as a wav-file. Only USVs produced during isolation and handling stages were used for further comparative acoustic analyses. The audible calls produced by subject animals during experimental trials were ignored during selection of audio files and calls for analysis and not measured.

### Acoustic analysis

Inspection of spectrograms and acoustic measurements have been conducted with Avisoft SASLab Pro software (Avisoft Bioacoustics, Berlin, Germany) and exported to Microsoft Excel (Microsoft Corp., Redmond, WA, USA). Following^14,32,39^, we defined the USV as frequency contour either continuous without breaks or with breaks shorter than 10 ms. If the separation break exceeded 10 ms, we considered that the contours belonged to two different calls. The USVs shorter 5 ms were ignored during the counting.

Inspection of spectrograms showed that only 14 of the 36 audio files (6 from males and 8 from females) contained at least 20 USVs at isolation stage and at least 20 USVs at handling stage. These 14 trials served for further acoustic analyses of joint and single USVs. Before acoustic measurements, all wav-files were subjected to 10 kHz high-pass filtering, to remove low-frequency noise. Preliminary analysis showed that USVs lacked in this frequency range. Spectrograms for analysis were created using sampling frequency 384 kHz Hamming window, fast Fourier transform (FFT) 1024 points, frame 50%, overlap 87.5%, providing frequency resolution 375 Hz and time resolution 0.33 ms.

Call rate. In each of the 14 trials, we labeled the start and end time of the isolation and handling stages basing on voice landmarks and calculated the stage duration as the end and start time difference. We counted the number of USVs per isolation and handling stages, and calculated call rate (USV/s) for each isolation and handling stage of the 14 trials, by dividing the number of USVs emitted at each stage to stage duration in seconds.

**Figure 1:**
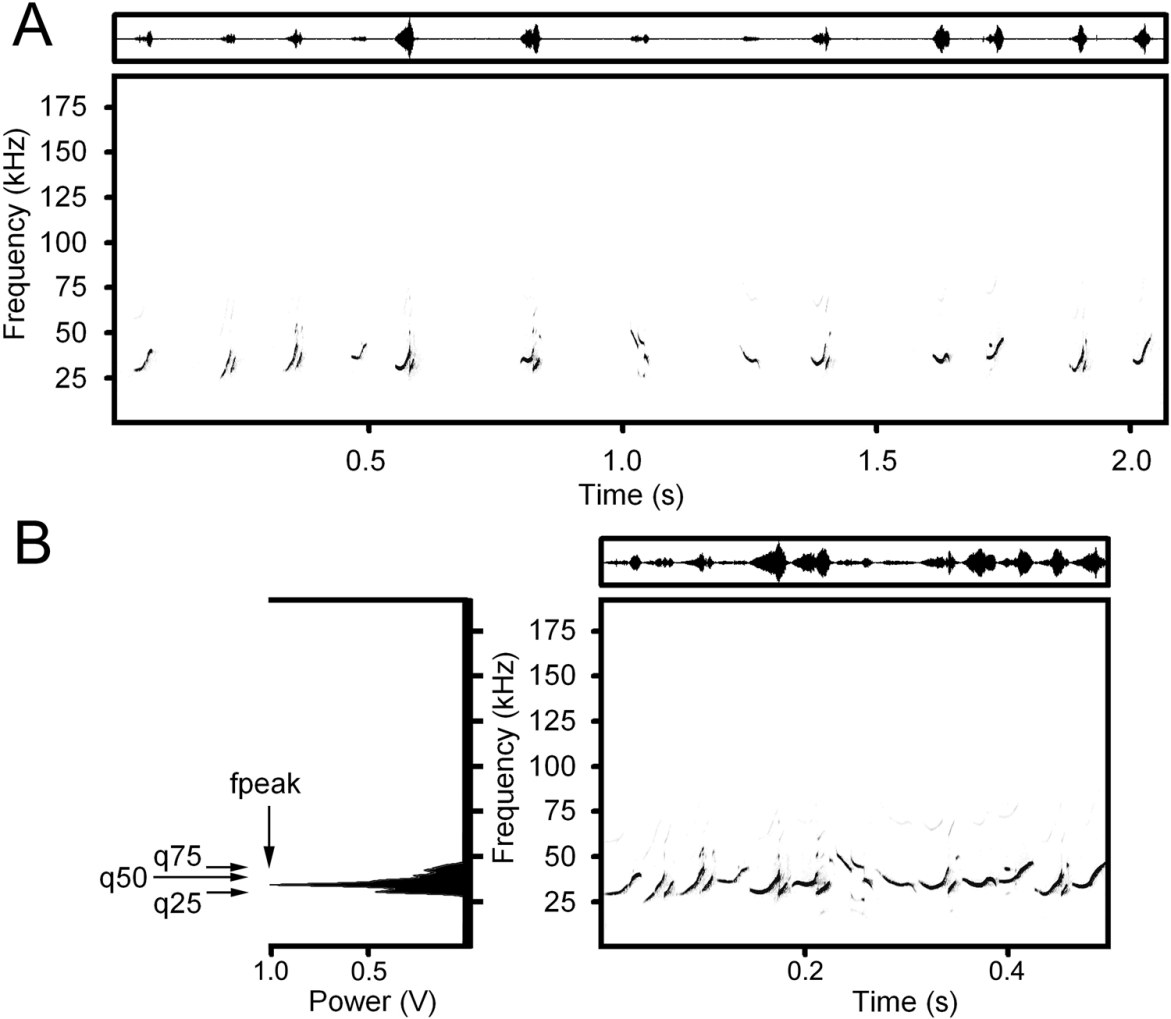
Procedure for preparation of a “joint call” and the maximum amplitude frequency and three power quartiles measure. (**A**) Spectrogram and wave-form of an intact natural sequence of USVs, separated with silent spaces, produced by adult female yellow steppe lemming. (**B**) Spectrogram, wave-form and power spectrum (left) of a part of the future joint call, made from the call sequence shown above. Measured acoustic variables: fpeak—the maximum amplitude frequency; q25—the lower quartile; q50—the medium quartile; q75—the upper quartile. Spectrogram was created using sampling frequency 192 kHz, Hamming window, FFT 1024 points, frame 50% and overlap 75%.

Measured variables for joint USVs. For the 14 trials, we prepared 28 “joint USVs” following^10^. For each isolation and handling stage we manually cut off the silent spaces between USVs, to prepare two joint USVs per trial. Using the automatic parameter measurement option of Avisoft, for each joint USV we measured the maximum amplitude frequency (fpeak) and three quartiles (q25, q50 and q75), covering respectively 25, 50 and 75% of call energy from the mean power spectrum of each joint call (Fig. 1 and Table S1). These call variables describe the relative distribution of energy over a call spectrum. The low values of the maximum amplitude frequency and of the quartiles reflect the shift of energy towards the lower frequencies, while the high values of these variables reflect the energy shift towards the higher frequencies.

Measured variables for single USVs. For each isolation and handling stage of the 14 trials, we selected for the detailed spectrographic analysis 20 USVs per stage, 40 USVs per animal (one animal provided only 39 USVs). As USVs following each other could be more similar, we, knowing the absolute number of USVs in each stage, selected for analysis USVs on the proportional basis. For example, if 60 USVs were present at this stage, we selected each third one: 1st, 4th, 7th, etc.; if 100 USVs were present, we selected each 5th USV: 1th, 6th, 11th, etc.; if 200 USVs were present, we selected each 10th USV: 1th, 11th, 21th, etc. If the selected call was damaged (due to the noise of animal transitions in setup), we selected for analysis a next call. In total, we included in analysis 559 USVs (279 USVs from isolation stage and 280 from handling stage).

**Figure 2:**
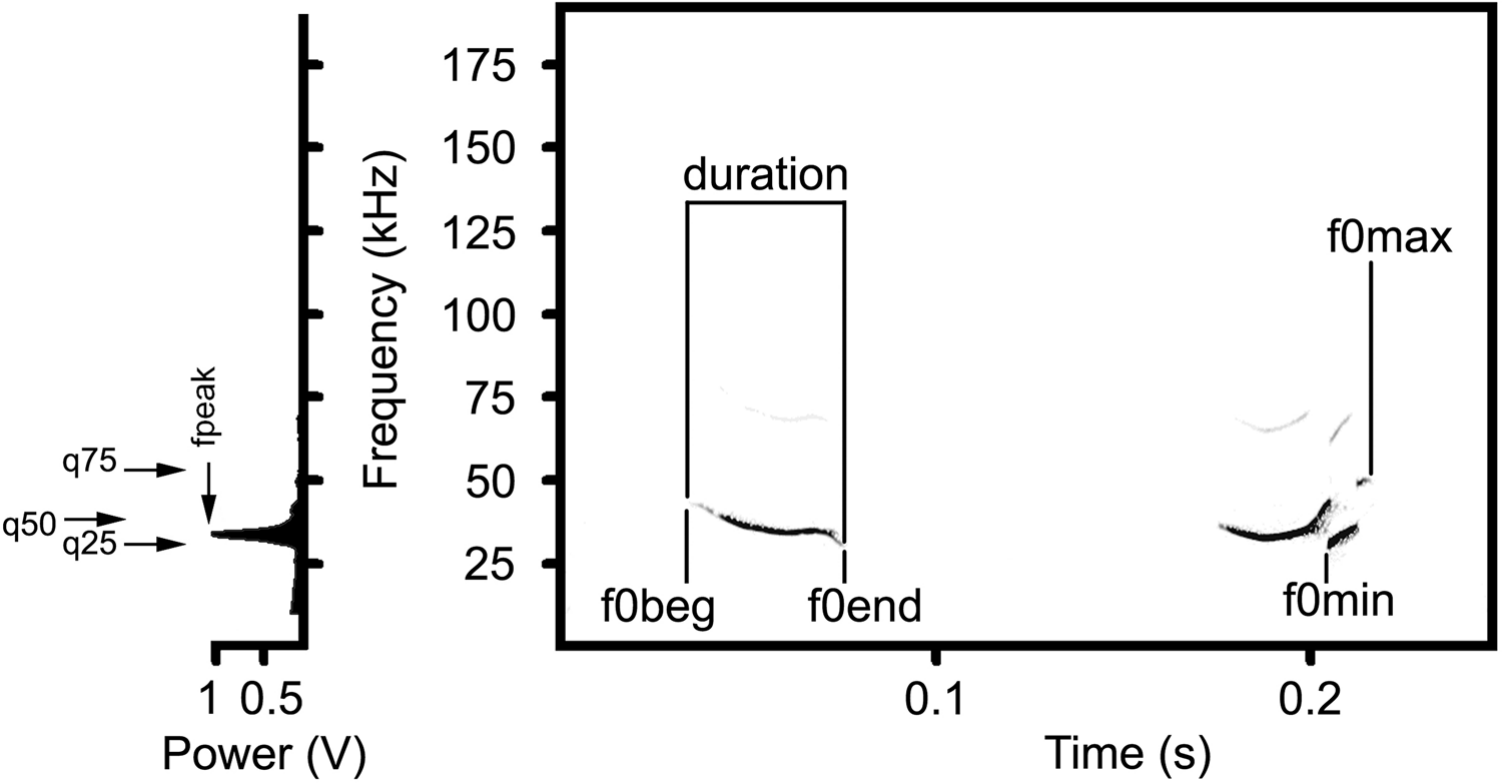
Measured variables for adult yellow steppe lemmings USV calls exemplified by a female USV without frequency jump and by a female USV with frequency jump. Spectrogram (right) and mean power spectrum of the first entire call (left). Designations: duration—call duration; f0beg—the fundamental frequency at the onset of a call; f0end—the fundamental frequency at the end of a call; f0max—the maximum fundamental frequency; f0min—the minimum fundamental frequency; fpeak—the frequency of maximum amplitude; q25—the lower quartile; q50—the medium quartile; q75—the upper quartile. Spectrogram was created using sampling frequency 192 kHz, Hamming window, FFT 1024 points, frame 50% and overlap 87.5%.

For each call, we measured, in the spectrogram window of Avisoft, the duration with the standard marker cursor, and the maximum fundamental frequency (f0max), the minimum fundamental frequency (f0min), the fundamental frequency at the onset of a call (f0beg), and the fundamental frequency at the end of a call (f0end) with the reticule cursor (Fig. 2 and Table S2). For each call, we measured, in the power spectrum window of Avisoft, the frequency of maximum amplitude (fpeak) from the call’s mean power spectrum and the three power quartiles (q25, q50, q75) of the entire call (Fig. 2 and Table S2). We calculated the depth of frequency modulation (df0) as the difference between f0max and f0min. In addition, for each call we scored the presence/absence of frequency jump (of 10 or more kHz up or down)^14,39,41,42^. Frequency jumps less than 10 kHz were not considered as frequency jumps.

### Statistics

All statistical analyses were carried out with STATISTICA (StatSoft, Inc., Tulsa, OK, USA). All tests were two-tailed and differences were considered significant where *p* < 0.05. Distributions of 57 measured parameter values of 70 distributions did not depart from normality (Kolmogorov–Smirnov test, *p* > 0.05). As ANOVA is relatively robust to departures from normality^43^, this was not an obstacle to the application of the parametric tests. We used a repeated measures ANOVA to compare USV call rates and the acoustics of joint USVs between isolation and handling stages. We used a two-way ANOVA with trial stage as fixed factor and animal ID as random factor, to compare the acoustics of USVs between isolation and handling stages.

### Disclaimer

The funder had no role in study design, data collection and analysis, decision to publish, or preparation of the manuscript.

### Ethical note

All animal experimentation was approved by the Committee of Bio-ethics of Lomonosov Moscow State University, research protocol # 2011-36. Experiments were performed in accordance with all applicable international, national, and/or institutional guidelines for the use of animals, including the ASAB/ABS Guidelines for the Use of Animals in Research. All experimental procedures followed the ARRIVE guidelines (https://arriveguidelines.org/arrive-guidelines).

## Supplementary Information

Supplementary Information 1.

Supplementary Information 2.

## Data Availability

All data generated or analysed during this study are included in this published article (and its Supplementary Information file).
